# Mobile phone use and stress, sleep disturbances, and symptoms of depression among young adults - a prospective cohort study

**DOI:** 10.1186/1471-2458-11-66

**Published:** 2011-01-31

**Authors:** Sara Thomée, Annika Härenstam, Mats Hagberg

**Affiliations:** 1 Occupational and Environmental Medicine, Department of Public Health and Community Medicine, University of Gothenburg, Gothenburg, Sweden; 2 Department of Work Science, University of Gothenburg, Gothenburg, Sweden

## Abstract

**Background:**

Because of the quick development and widespread use of mobile phones, and their vast effect on communication and interactions, it is important to study possible negative health effects of mobile phone exposure. The overall aim of this study was to investigate whether there are associations between psychosocial aspects of mobile phone use and mental health symptoms in a prospective cohort of young adults.

**Methods:**

The study group consisted of young adults 20-24 years old (n = 4156), who responded to a questionnaire at baseline and 1-year follow-up. Mobile phone exposure variables included frequency of use, but also more qualitative variables: demands on availability, perceived stressfulness of accessibility, being awakened at night by the mobile phone, and personal overuse of the mobile phone. Mental health outcomes included current stress, sleep disorders, and symptoms of depression. Prevalence ratios (PRs) were calculated for cross-sectional and prospective associations between exposure variables and mental health outcomes for men and women separately.

**Results:**

There were cross-sectional associations between *high *compared to *low mobile phone use *and stress, sleep disturbances, and symptoms of depression for the men and women. When excluding respondents reporting mental health symptoms at baseline, *high mobile phone use *was associated with sleep disturbances and symptoms of depression for the men and symptoms of depression for the women at 1-year follow-up. All qualitative variables had cross-sectional associations with mental health outcomes. In prospective analysis, *overuse *was associated with stress and sleep disturbances for women, and *high accessibility stress *was associated with stress, sleep disturbances, and symptoms of depression for both men and women.

**Conclusions:**

High frequency of mobile phone use at baseline was a risk factor for mental health outcomes at 1-year follow-up among the young adults. The risk for reporting mental health symptoms at follow-up was greatest among those who had perceived accessibility via mobile phones to be stressful. Public health prevention strategies focusing on attitudes could include information and advice, helping young adults to set limits for their own and others' accessibility.

## Background

Mental health problems have been increasing among young people in Sweden and around the world [1,2]. Cultural and social changes in terms of increased materialism and individualism have been discussed in relation to this [3,4], including the possibility of a decreasing stigma about mental illness, improved screening for mental illness, and increased help-seeking behaviors [5]. Because of the quick development and widespread use of mobile phones, and their vast effect on communication and interactions in work and private life, it is important to study possible negative health effects of the exposure. Extensive focus has been on exposure to electromagnetic fields (EMF). Self-reported symptoms associated with using mobile phones most commonly include headaches, earache, and warmth sensations [6,7], and sometimes also perceived concentration difficulties and fatigue [6]. However, EMF exposure due to mobile phone use is not currently known to have any major health effects [8]. Another aspect of exposure is ergonomics. Musculoskeletal symptoms due to intensive texting on a mobile phone have been reported [9], and techniques used for text entering have been studied in connection with developing musculoskeletal symptoms [10]. However, our perspective is predominantly psychosocial.

In a previous study we found prospective associations between high information and communications technology (ICT) use, including high frequency of mobile phone use, and reported mental health symptoms among young adult college and university students [11], but concluded that the causal mechanisms are unclear. The study was followed by a qualitative interview study with 32 subjects with high computer or mobile phone use, who had reported mental health symptoms at 1-year follow-up. Based on the young adults' own perceptions and ideas of associations, a model of possible paths for associations between ICT use and mental health symptoms was proposed [12], with pathways to stress, depression, and sleep disorders via the consequences of high quantitative ICT use, negative quality of use, and user problems. Central factors appearing to explain high quantitative use were personal dependency, and demands for achievement and availability originating from domains of work, study, the social network, and the individual's own aspirations. These factors were also perceived as direct sources of stress and mental health symptoms. Consequences of high quantitative mobile phone exposure included mental overload, disturbed sleep, the feeling of never being free, role conflicts, and feelings of guilt due to inability to return all calls and messages. Furthermore, addiction or dependency was an area of concern, as was worry about possible hazards associated with exposure to electromagnetic fields. For several participants in the study, however, a major stressor was to not be available. The study concluded that there are many factors in different domains that should be taken into consideration in epidemiological studies concerning associations between ICT use and mental health symptoms [12].

Based on the previous studies, we wanted to focus on some aspects of mobile phone exposure other than mere quantity of use. For example, demands on being available or reachable, regardless of time and space, could be argued to be a stressor irrespective of actual frequency of use. Another key determinant may be the extent to which a person actually perceives his or her own accessibility as stressful. Furthermore, accessibility implies the possibility to be disturbed at all hours, even at nighttime. Having one's sleep interrupted repeatedly can have direct effects on recovery and health. In a study among Finnish adolescents, intensive mobile phone use was linked to poor perceived health among girls, both directly and through poor sleep and waking-time tiredness [13]. Another area of concern could be addiction to the mobile phone. Intensive mobile phone use has been associated with dependency on the mobile phone [14,15], and *problematic mobile phone use *has been a focus in the literature concerning psychological aspects of mobile phone use, where criteria for substance addiction diagnoses or behavioral addictions [16,17] have been used to define problematic use [18-24] including compulsive short messaging service (SMS) use [20]. In this context, heavy or problem mobile phone use (overuse) has been associated with somatic complaints, anxiety, and insomnia [21], depression [21,24], psychological distress [22], and an unhealthy lifestyle [25]. However, possible positive effects of mobile phone use on mental health can also be hypothesized, for instance the ease of reaching someone to talk to when in need, implying access to social support. Social support buffers negative effects of stress [26], while low social support is a risk factor associated with mental health symptoms [27].

We have previously studied ICT use in relation to mental health symptoms among highly selected study groups (college and university students studying medicine and information technology) [11,12]. Most investigations we have found on mobile phone use and mental health outcomes have been cross-sectional studies performed among mainly college students (e.g., [15,19-23]). It is important to examine possible associations between mobile phone use and mental health outcomes also in a more general or heterogeneous population of young adults, using a longitudinal design.

### Aims

The overall aim of this study was to investigate whether there are associations between psychosocial aspects of mobile phone use and mental health symptoms in a prospective cohort of young adults. Specific aims were to examine whether the frequency of mobile phone use, but also more qualitative aspects of mobile phone use (demands on availability, perceived stressfulness of accessibility, being awakened at night by the mobile phone, and perceived personal overuse of the mobile phone), are associated with reported stress, symptoms of depression, and sleep disturbances. Furthermore, we wanted to examine whether frequency of mobile phone use is associated with perceived social support.

## Methods

### Study population and data collection

**Figure 1:**
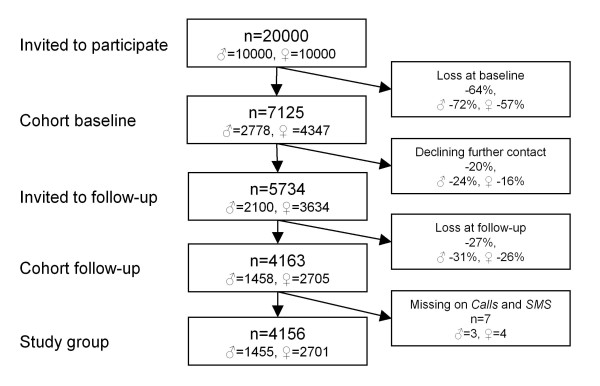
**Participation process**. The participation process from study population to study group

The study population consisted of a cohort of young adults (Figure 1), 20-24 years old (corresponding to the United Nations' definition of young adults [28]). Ten thousand men and 10 000 women, born between 1983 and 1987, were randomly selected from the general population (from a registry held by the Swedish Tax Agency), 50% living in the County of Västra Götaland, Sweden, and 50% in the rest of the country. In October 2007, a questionnaire containing questions about health, work, and leisure-related exposure factors, background factors, and psychosocial factors was sent by post to the selected population [29]. Besides returning the postal questionnaire it was also possible to respond to the questionnaire via the web if desired. A lottery ticket (value approx. 1 Euro) was attached to the cover letter and could be used whether the recipient participated in the study or not. Two reminders were sent by post. The response rate at baseline was 36% (n = 7125). One year later, those respondents who had indicated that they would accept to be offered to participate in further studies (n = 5734) were invited to respond to an identical questionnaire, this time administered via the web. The data collection process was otherwise similar to that at baseline, but with the addition of a third reminder offering a paper version of the questionnaire and two cinema tickets to respondents. The response rate at follow-up was 73% (n = 4163). After excluding those who failed to respond to both questions concerning frequency of mobile phone and SMS use at baseline, 4156 remained in the study group. All in all, non-participation and dropout from the study was 79% (Figure 1).

### Mobile phone exposure variables

Information about mobile phone exposure was collected from the baseline questionnaire. This included the average number of mobile phone calls made and received, and of SMS messages sent and received, per day, but also more qualitative aspects of mobile phone use, including how often the respondent was awakened at night by the mobile phone, how he or she perceived demands on availability, and whether he or she perceived the accessibility via mobile phones to be stressful, as well as perceptions regarding personal overuse of the mobile phone. Responses were divided into *high*, *medium*, and *low *categories, based on the frequency distribution of responses, except for *overuse *which was categorized according to number of items confirmed. A combined quantitative mobile phone use variable was constructed by merging the variables *frequency of calls *and *frequency of SMS messages *(Spearman correlation r = 0.35, p < 0.0001). The *mobile phone use *variable correlated well with the original *calls *and *SMS *variables (r = 0.73, p <.0001, and r = 0.84, p <. 0001, respectively).

Mobile phone variables, questionnaire items, response categories, and response classifications are presented in Table 1.

**Table 1:** Mobile phone variables at baseline

Category	Mobile phone variables	Men		Women	
		n = 1455		n = 2701	
		Freq	%	Freq	%
	**Frequency of calls**				
Low	0 per day	69	5	51	2
Low	1-5 per day	952	65	1946	72
Med	6-10 per day	301	21	543	20
High	11-20 per day	97	7	108	4
High	More than 20 per day	36	2	47	2
	**Frequency of SMS messages**				
Low	0 per day	126	9	58	2
Low	1-5 per day	906	62	1609	60
Med	6-10 per day	262	18	634	23
High	11-20 per day	98	7	259	10
High	More than 20 per day	60	4	140	5
	**Mobile phone use**				
Low	Low Calls + Low SMS	804	55	1433	53
Med	Low Calls + Med SMS or vice versa	326	22	616	23
High	High Calls and/or High SMS, or Med Calls + Med SMS	323	22	645	24
	**Awakened at night**				
Low	Never	600	41	989	37
Med	Only occasionally	657	45	1248	46
High	A few times per month	164	11	386	14
High	A few times per week	27	2	68	3
High	Almost every day	6	0	9	0
	**Availability demands**				
Low	Never	23	2	12	0
Low	Now and then, but not daily	82	6	86	3
Low	Daily, but not all day	278	19	828	31
Med	All day	680	47	1127	42
High	Around the clock	388	27	642	24
	**Accessibility stress**				
Low	Not at all stressful	892	61	1229	46
Med	A little bit stressful	418	29	1083	40
High	Rather stressful	115	8	311	12
High	Very stressful	28	2	75	3
	**Overuse**				
	Item 1: Use too much	184	13	587	22
	Item 2: Tried to cut down unsuccessfully	87	6	371	14
Low	No item	1199	84	1898	71
Med	One item	183	13	579	22
High	Both items	41	3	187	7

Frequencies (Freq) and percentages (%) in mobile phone variables for the men and women, including categorizations into Low, Medium (Med), and High. Questionnaire items are presented in footnote^1^. Missing values (non-responses to items) are not accounted for, which means that the *n *varies for the variables.

^1^Questionnaire items; Frequency of calls: *How many mobile phone calls on average have you made and received per day (the past 30 days)*?, Frequency of SMS messages: *How many SMS messages on average have you sent and received per day (the past 30 days)?*, Awakened at night: *How often have you been awakened by the mobile phone at night (the past 30 days)?*, Availability demands: *To what extent are you expected by those around you to be accessible via the mobile phone?*, Accessibility stress: *To what extent do you perceive accessibility via mobile phones as stressful?*, Overuse: *1. Do you or someone close to you think that you use the mobile phone too much?, 2. Have you tried, but failed, to cut down on your use of the mobile phone?*

### Mental health outcome variables

Information about mental health symptoms was collected from the cohort study questionnaire at baseline and at follow-up.

The outcome variable *Current stress *was constituted by a validated single-item stress-indicator [30]: *Stress means a situation when a person feels tense, restless, nervous, or anxious or is unable to sleep at night because his/her mind is troubled all the time. Are you currently experiencing this kind of stress? *Response categories were: a = *not at all*, b = *just a little*, c = *to some extent*, d = *rather much*, e = *very much*. The responses were divided into *Yes *(responses d-e) and *No *(responses a-c), based on frequency distribution while yet taking content of response categories into account.

The *Sleep disturbances *variable was constructed by including the most common sleep disorders (insomnia, fragmented sleep and premature awakening) into a single-item, adapted from the The Karolinska Sleep Questionnaire [31]: *How often have you had problems with your sleep these past 30 days (e.g., difficulties falling asleep, repeated awakenings, waking up too early)? *Response categories were: a = *never*, b = *a few times per month*, c = *several times per week*, and d = *every day*. The responses were divided into *Yes *(responses c-d) and *No *(responses a-b), based on clinical significance.

*Symptoms of depression (one item) *and *symptoms of depression (two items) *were made up by the two depressive items from the Prime-MD screening form [32]: *During the past month, have you often been bothered by: *(a) *little interest or pleasure in doing things? *(b) *feeling down, depressed, or hopeless? *Response categories were *Yes *and *No*. It is proposed that it is sufficient if one of the two items is confirmed in screening to go forward with clinical assessment of mood disorder. This procedure has high sensitivity for major depression diagnosis in primary care populations [32,33]. In our cohort study group, approximately 50% of the men and almost 65% of the women confirmed at least one of the two depressive items, which indicates that the instrument is probably very sensitive but has low specificity in our study group. Therefore, we constructed two outcomes: *Symptoms of depression (one item)*, in which the *Yes *category contained those who confirmed only one of the depressive items, and *Symptoms of depression (two items)*, in which the *Yes *category contained those who confirmed both depressive items. The *No *category in both outcomes contained those who disclaimed the two depressive items.

### Background factors and social support

Background factors were collected to describe the study group and to adjust for in the multivariate analysis, including: relationship status: *single *or *in a relationship*; highest completed educational level: *elementary school *(basic schooling for 6-16-year-olds), *upper secondary school*, or *college or university studies*; and occupation: *working, studying*, or *other *(*other *included being on long-term sick leave, or on parental or other leave, or being unemployed). The variable *social support *was based on the item: *When I have problems in my private life I have access to support and help*, a one-item adaptation of the social support scale in the Karasek-Theorell job content questionnaire [34], here relating to private life (rather than work life). Response categories were: a = *applies very poorly*; b = *applies rather poorly*; c = *applies rather well*; d = *applies very well*. The responses were categorized as *low *(response categories a and b), *medium *(response category c), and *high *(response category d).

### Analysis

All analyses were performed using the statistical software package SAS, version 9.2 (SAS Institute, Cary, NC, USA). Spearman correlation analysis was used to examine associations between the mobile phone exposure variables, and between mobile phone use and social support. The Cox proportional hazard model (PHREG proc with time set to 1) was used to calculate prevalence ratios (PRs) with a 95% confidence interval (CI) for multivariate analysis of cross-sectional and prospective associations between exposure variables and mental health outcomes. The robust variance option (COVS) was used in the cross-sectional analysis to produce adequate CIs [35,36]. The *low *category in each exposure variable was used as reference level. The PRs were adjusted for background factors including relationship status, educational level, and occupation at baseline. Missing values (non-responses to items) were excluded from the analyses, which means that the *n *varied in the analyses. Prevalence ratios with a CI not including 1.00 (before round-off) were considered statistically significant. In the prospective analysis, subjects who reported symptoms at baseline were excluded from the analysis of the mental health outcome variable concerned. All analyses were done separately for the men and women.

The study was approved by the Regional Ethics Review Board in Gothenburg, Sweden (Reg. no. 191-05).

## Results

### Study group characteristics

Fifty-two percent of the men and 34% of the women were single at baseline. A majority of the respondents had completed upper secondary school, 13% of the men and 16% of the women had finished college or university studies, while 5% of the men and 6% of the women only elementary school. Fifty-one percent of the men and 41% of the women reported work as main occupation, while 40% of the men and 48% of the women studied, and 8% of the men and 12% women were categorized as *other*. Forty-three percent of the men and 56% of the women reported high social support, 41% of the men and 32% of the women reported medium social support, and 16% of the men and 13% of the women reported low social support.

A little more than half of the participants were categorized as having *low mobile phone use *(five or fewer calls and five or fewer SMS messages per day) and 22% of the men and 24% of the women as having *high use *(eleven or more calls or SMS messages per day) (Table 1). A massive majority reported that they were expected to be available on a daily basis and one out of four around the clock. Only a few percent found accessibility via mobile phones very stressful, while about half of the participants did not find it stressful at all. Most participants were never, or only on rare occasions, woken up by the mobile phone, and only a few reported being woken by the mobile phone on a weekly basis. Thirteen percent of the men and 22% of the women indicated that they themselves, or someone close to them, thought that they used the mobile phone too much, and 6 and 14%, respectively, had tried, but failed, to cut down on mobile phone use (Table 1).

The women reported stress almost twice as often as the men (29% compared to 16%) at baseline. Twenty-three percent of the men and 34% of the women indicated sleep disturbances. Of the men, 27% reported one and 24% two symptoms of depression, and of the women, 30% reported one and 34% two symptoms of depression. Among participants who were symptom-free at baseline (in outcome variable concerned), the prevalence at 1-year follow-up was as follows for the men and women, respectively; *current stress: *10% and 19%, *sleep disturbances: *15% and 20%, *symptoms of depression (one item): *24% and 28%, and *symptoms of depression (two items): *12% and 18%.

### Drop-out analysis

The drop-out group at the initial cohort baseline consisted of more men (a difference of 17 percentage points), were somewhat younger (an age difference of <0.1 years), more often married (a difference of 1.4 percentage points), and more often foreign-born (8 percentage points), compared to the study population invited to participate [29]. The final study group (n = 4156) consisted of almost twice as many women as men (65% vs. 35%). Compared to the initial cohort baseline (n = 7125), the study group participants were slightly less often single (40% compared to 42%), had a slightly higher educational level (with 15% compared to 14% having college or university level education, and 5% compared to 7% having completed only elementary school), and were less often working (44% compared to 48%) and more often studying (45% compared to 41%) at baseline. The level of mobile phone use was slightly lower in the study group; 54% were categorized as low mobile phone users compared to 51% in the initial cohort baseline, while 23% compared to 26% were categorized as frequent (high) mobile phone users.

### Associations between the mobile phone variables at baseline

The frequency of mobile phone use variable had low positive correlations with all of the more qualitative mobile phone variables using Spearman correlation analysis (see Table 2). Furthermore, there were low positive (or little if any) associations between most qualitative mobile phone variables, and no association between *availability demands *and *accessibility stress*.

**Table 2:** Correlations between the mobile phone exposure variables at baseline

	Awakened at night	Availability demands	Accessibility stress	Overuse
	Men/Women	Men/Women	Men/Women	Men/Women
**Mobile phone use**	0.31^a^/0.32^a^	0.24^a^/0.23^a^	0.09^b^/0.10^a^	0.24^a^/0.30^a^
**Awakened at night**		0.28^a^/0.28^a^	0.07^c^/0.09^a^	0.14^a^/0.21^a^
**Availability demands**			-0.002 ns/0.03 ns	0.10^a^/0.11^a^
**Accessibility stress**				0.20^a^/0.22^a^

Spearman correlation coefficients for the men (n = 1455) and women (n = 2701). All correlations are statistically significant (^a^p < 0.001, ^b^p < 0.01, ^c^p < 0.05) unless indicated as non-significant (ns).

### Mobile phone use and social support

Frequency of mobile phone use had little if any association with perceived access to social support for the men (r = 0.08, p < 0.01) and no association for the women (r = -0.01, p = 0.48).

### Cross-sectional associations between mobile phone variables and mental health outcomes at baseline

There were positive associations between *high *compared to *low mobile phone use *and current stress, sleep disturbances, and symptoms of depression (two items) for both the men and the women, after adjusting for relationship status, educational level, and present occupation (Table 3). Among the more qualitative mobile phone variables, *availability demands *was associated with current stress and symptoms of depression (two items) for the men and with all mental health outcomes for the women. *Being awakened at night *was associated with current stress, sleep disturbances, and symptoms of depression (one and two items) for the men and women. For the men, *overuse *was associated with current stress, sleep disturbances, and symptoms of depression (two items), and for the women, *overuse *was associated with all mental health outcomes. The strongest associations (highest PRs) were found for *accessibility stress *in relation to the mental health outcomes. For the men, *accessibility stress *was associated with current stress and symptoms of depression (one and two items), and for the women, *accessibility stress *was associated with all mental health outcomes.

**Table 3:** Associations between mobile phone variables and mental health outcomes at baseline for men (n = 1455) and women (n = 2701)

		CURRENT STRESS				SLEEP DISTURBANCES				SYMPTOMS OF DEPRESSION One item				SYMPTOMS OF DEPRESSION Two items			
		n	Prev %	PR	95% CI	n	Prev %	PR	95% CI	n	Prev %	PR	95% CI	n	Prev %	PR	95% CI
**Mobile phone use**																	
**Men**	High	295	23	**1.9**	**1.42-2.54**	294	33	**1.7**	**1.40-2.19**	208	37	1.2	0.94-1.46	215	39	**1.3**	**1.02-1.58**
	Medium	309	16	1.3	0.98-1.84	309	21	1.1	0.87-1.47	235	39	1.2	1.00-1.48	216	34	1.1	0.90-1.43
	Low	749	13	1.0		746	20	1.0		573	34	1.0		551	31	1.0	
**Women**	High	570	32	**1.2**	**1.07-1.45**	566	43	**1.4**	**1.21-1.56**	351	51	**1.2**	**1.04-1.35**	384	55	**1.2**	**1.06-1.34**
	Medium	559	31	**1.2**	**1.06-1.44**	554	34	1.1	0.98-1.31	366	45	1.1	0.93-1.22	390	49	1.1	0.98-1.26
	Low	1304	26	1.0		1300	30	1.0		898	42	1.0		916	44	1.0	
**Availability demands**																	
**Men**	High	360	18	**1.5**	**1.04-2.15**	359	27	1.3	0.96-1.64	251	35	1.0	0.76-1.20	267	39	**1.3**	**1.00-1.64**
	Medium	634	17	**1.5**	**1.04-2.02**	631	21	1.1	0.82-1.36	485	34	0.9	0.74-1.09	468	31	1.1	0.86-1.38
	Low	357	12	1.0		357	21	1.0		280	39	1.0		245	30	1.0	
**Women**	High	586	33	**1.3**	**1.14-1.57**	583	41	**1.4**	**1.21-1.61**	356	47	1.2	0.99-1.33	413	54	**1.3**	**1.10-1.43**
	Medium	1006	28	1.1	0.95-1.29	999	34	**1.2**	**1.03-1.36**	667	47	**1.2**	**1.02-1.31**	683	49	**1.2**	**1.02-1.31**
	Low	841	25	1.0		838	28	1.0		591	41	1.0		594	41	1.0	
**Awakened at night**																	
**Men**	High	182	24	**1.8**	**1.29-2.51**	182	35	**1.9**	**1.44-2.43**	117	44	**1.3**	**1.05-1.72**	129	49	**1.6**	**1.27-2.03**
	Medium	613	16	1.2	0.90-1.58	610	23	**1.3**	**1.04-1.64**	465	36	1.1	0.93-1.33	441	32	1.1	0.92-1.37
	Low	560	13	1.0		559	18	1.0		436	33	1.0		413	29	1.0	
**Women**	High	417	36	**1.5**	**1.24-1.75**	413	44	**1.4**	**1.24-1.67**	234	51	1.1	0.98-1.34	294	61	**1.4**	**1.26-1.65**
	Medium	1118	29	**1.2**	**1.04-1.40**	1111	33	1.1	0.97-1.26	735	44	1.0	0.90-1.14	786	48	**1.2**	**1.03-1.32**
	Low	901	24	1.0		900	30	1.0		646	44	1.0		614	41	1.0	
**Accessibility stress**																	
**Men**	High	131	39	**3.5**	**2.58-4.64**	131	27	1.3	0.98-1.81	71	54	**1.8**	**1.42-2.31**	91	64	**2.4**	**1.96-2.94**
	Medium	387	18	**1.6**	**1.21-2.14**	385	25	1.2	0.99-1.53	287	41	**1.3**	**1.12-1.60**	268	37	**1.4**	**1.20-1.67**
	Low	835	11	1.0		833	21	1.0		658	31	1.0		623	27	1.0	
**Women**	High	345	49	**2.5**	**2.13-2.94**	345	47	**1.6**	**1.39-1.85**	199	59	**1.4**	**1.24-1.65**	224	64	**1.7**	**1.46-1.90**
	Medium	986	31	**1.6**	**1.38-1.87**	978	34	**1.2**	**1.03-1.32**	610	44	1.1	0.94-1.20	707	52	**1.4**	**1.22-1.54**
	Low	1104	20	1.0		1100	29	1.0		807	42	1.0		761	38	1.0	
**Overuse**																	
**Men**	High	38	32	**2.1**	**1.30-3.50**	38	37	**1.7**	**1.10-2.55**	23	43	1.3	0.83-2.15	28	54	**1.7**	**1.18-2.41**
	Medium	170	17	1.2	0.81-1.67	170	23	1.1	0.79-1.43	117	38	1.1	0.87-1.44	126	42	**1.4**	**1.10-1.72**
	Low	1120	15	1.0		1116	22	1.0		856	35	1.0		809	31	1.0	
**Women**	High	165	41	**1.6**	**1.31-1.96**	164	41	**1.3**	**1.10-1.61**	93	54	**1.3**	**1.04-1.55**	114	62	**1.4**	**1.23-1.67**
	Medium	526	33	**1.3**	**1.09-1.46**	522	38	**1.2**	**1.04-1.35**	328	52	**1.2**	**1.06-1.36**	355	55	**1.2**	**1.10-1.38**
	Low	1716	26	1.0		1709	32	1.0		1179	42	1.0		1204	44	1.0	

Prevalence (prev %) of mental health symptoms in each exposure category is shown. The prevalence ratios (PRs) with 95% confidence intervals (CIs) were adjusted for relationship status, educational level, and occupation. Missing values (non-responses to items) were excluded from the analyses, which means that the *n *varied in the analyses. Prevalence ratios with a CI not including 1.00 (before round-off) are given in bold.

In all cross-sectional analyses, the high category of the exposure variables generated a higher or equivalent PR compared to the medium category, indicating a dose-response relationship between the exposure variables and mental health outcomes, though not all associations were statistically significant. All but three PRs (77/80) were greater than 1.0.

### Prospective associations between mobile phone variables at baseline and mental health outcomes at 1-year follow-up

When excluding participants reporting symptoms at baseline from the analysis of the outcome variable concerned, *high *compared to *low mobile phone use *at baseline was associated with reported sleep disturbances and symptoms of depression (one item) in the men (PR 1.8, CI 1.21-2.69 and PR 1.7, CI 1.14-2.46, respectively) and symptoms of depression (two items) in the women (PR 1.5, CI 1.02-2.24), at 1-year follow-up (Table 4).

**Table 4:** Prospective associations between mobile phone variables at baseline and mental health outcomes at 1-year follow-up

		CURRENT STRESS				SLEEP DISTURBANCES				SYMPTOMS OF DEPRESSION One item				SYMPTOMS OF DEPRESSION Two items			
		n	Prev %	PR	95% CI	n	Prev %	PR	95% CI	n	Prev %	PR	95% CI	n	Prev %	PR	95% CI
**Mobile phone use**																	
**Men**	High	227	8	0.9	0.51-1.47	193	21	**1.8**	**1.21-2.69**	120	38	**1.7**	**1.14-2.46**	86	13	1.1	0.53-2.10
	Medium	258	11	1.2	0.76-1.87	243	17	1.4	0.98-2.11	121	30	1.4	0.94-2.10	105	19	1.5	0.86-2.53
	Low	652	10	1.0		596	13	1.0		333	23	1.0		305	15	1.0	
**Women**	High	389	20	1.1	0.84-1.43	323	24	1.2	0.91-1.57	131	39	1.2	0.88-1.69	120	33	**1.5**	**1.02-2.24**
	Medium	382	18	1.0	0.75-1.30	367	20	1.1	0.81-1.40	161	31	0.9	0.68-1.30	150	26	1.2	0.83-1.79
	Low	968	19	1.0		909	19	1.0		435	33	1.0		370	22	1.0	
**Availability demands**																	
**Men**	High	296	14	1.6	0.97-2.56	261	19	1.4	0.91-2.12	137	30	1.2	0.76-1.85	120	20	1.6	0.88-3.05
	Medium	525	8	0.9	0.56-1.44	491	14	1.0	0.66-1.45	285	26	1.0	0.70-1.52	246	15	1.1	0.63-1.96
	Low	314	9	1.0		280	15	1.0		152	26	1.0		131	14	1.0	
**Women**	High	390	22	1.3	0.99-1.76	342	22	1.1	0.81-1.45	146	32	0.9	0.62-1.23	141	30	1.4	0.94-2.17
	Medium	722	19	1.1	0.87-1.45	655	20	1.0	0.79-1.30	281	31	0.8	0.63-1.12	263	26	1.2	0.85-1.78
	Low	625	17	1.0		599	19	1.0		299	37	1.0		235	20	1.0	
**Awakened at night**																	
**Men**	High	138	13	1.4	0.80-2.42	116	21	1.4	0.90-2.31	57	32	1.1	0.68-1.94	48	19	1.4	0.65-2.86
	Medium	516	10	1.1	0.71-1.60	462	15	1.0	0.74-1.47	264	27	1.0	0.74-1.45	225	15	1.0	0.63-1.65
	Low	485	9	1.0		456	15	1.0		254	26	1.0		224	16	1.0	
**Women**	High	268	22	1.2	0.86-1.60	229	23	1.2	0.87-1.68	96	39	1.2	0.84-1.81	78	24	1.1	0.62-1.80
	Medium	791	17	0.9	0.70-1.12	740	20	1.1	0.86-1.39	324	33	1.0	0.78-1.34	299	28	1.3	0.90-1.77
	Low	680	20	1.0		630	19	1.0		307	33	1.0		263	22	1.0	
**Accessibility stress**																	
**Men**	High	80	19	**2.2**	**1.22-3.80**	94	23	**1.7**	**1.06-2.71**	25	24	0.9	0.40-2.14	27	30	**2.3**	**1.06.4.98**
	Medium	317	11	1.3	0.88-2.01	288	16	1.2	0.84-1.69	145	32	1.3	0.89-1.79	123	20	1.6	0.96-2.56
	Low	740	9	1.0		651	14	1.0		404	25	1.0		347	13	1.0	
**Women**	High	176	32	**2.2**	**1.61-3.00**	181	27	**1.5**	**1.10-2.14**	54	43	1.3	0.82-2.03	58	47	**2.4**	**1.50-3.68**
	Medium	677	21	**1.5**	**1.15-1.85**	645	21	1.2	0.96-1.54	280	35	1.1	0.84-1.42	241	24	1.2	0.82-1.63
	Low	885	15	1.0		773	18	1.0		393	32	1.0		341	21	1.0	
**Over-use**																	
**Men**	High	26	12	1.2	0.36-3.71	23	22	1.4	0.58.3.49	11	54	1.9	0.82-4.45	7	29	2.0	0.48-8.43
	Medium	141	13	1.3	0.79-2.18	129	13	0.9	0.52-1.43	67	28	1.1	0.68-1.80	53	9	0.6	0.25-1.55
	Low	953	10	1.0		866	16	1.0		484	26	1.0		428	16	1.0	
**Women**	High	98	21	1.3	0.80-1.97	94	32	**1.8**	**1.21-2.62**	35	29	0.9	0.46-1.63	33	24	1.1	0.51-2.16
	Medium	353	24	**1.4**	**1.08-1.79**	323	24	**1.4**	**1.06-1.78**	125	40	1.2	0.89-1.66	109	31	1.4	0.92-2.00
	Low	1269	17	1.0		1163	18	1.0		560	33	1.0		491	23	1.0	

Participants who reported symptoms at baseline were excluded from prospective analysis of mental health outcome concerned. Study group *n *in prospective analysis was for *Current stress*: 1222 men and 1913 women, *Sleep disturbances*: 1107 men and 1762 women, *Symptoms of depression (one item)*: 617 men and 791 women, and *Symptoms of depression (two items)*: 534 men and 692 women. Prevalence (prev %) of mental health symptoms at 1-year follow-up in each exposure category is shown. The prevalence ratios (PRs) with 95% confidence intervals (CIs) were adjusted for relationship status, educational level and occupation. Missing values (non-responses to items) were excluded from the analyses, which means that the *n *varied in the analyses. Prevalence ratios with a CI not including 1.00 are given in bold.

There were no clear associations between *availability demands *or *being awakened at night *and the mental health outcomes. For women, *medium overuse *was associated with current stress and *high *and *medium overuse *was associated with sleep disturbances. *High accessibility stress *was associated with current stress, sleep disturbances, and symptoms of depression (two items) for both the men and the women. In the majority of analyses (32/40), the *high *category of the exposure variable generated a higher PR compared to the *medium *category.

## Discussion

Frequent mobile phone use was associated with current stress, sleep disturbances, and symptoms of depression among the young adult men and women in cross-sectional analysis. Prospective analysis indicated that high frequency of mobile phone use could be a risk factor (or marker) for developing sleep disturbances in the men, and symptoms of depression in both the men and women, at 1-year follow-up. The pattern of PRs larger than 1.0 was rather consistent (though not all statistically significant), suggesting a robustness of results, and there was even an indication towards a dose-response relationship between exposure and mental health outcomes (if looking only at PRs). It should be noted that the "high" category of mobile phone use in our study does not reflect an extreme part of the population, since almost 25% of the study group belonged to this category. The use of the Cox regression procedure for estimating PRs gives wider than adequate CIs [35], which was corrected for in the cross-sectional analysis by adding the robust variance option. However, in the prospective analysis the CIs are still conservative. The results are further supported by the finding of prospective associations between high frequency of mobile phone use and mental health outcomes in our previous study among young adult university students [11].

The majority of the young adults reported that they were expected to be reachable via the mobile phone all day or around the clock. One could expect that this would feel compelling and perhaps even stressful, but most respondents did not consider the accessibility to be stressful, and there was no association between the two variables. Yet, expected availability around the clock was associated with most mental health outcomes in cross-sectional analysis (no clear prospective associations). The risk for reporting mental health symptoms at follow-up was greatest among those respondents who had indicated that they perceived the accessibility to be *rather *or *very stressful*, and in cross-sectional analysis, it was even sufficient to consider the accessibility to be just *a little stressful *for higher prevalence of mental health outcomes. The over-all low associations between the mobile phone variables suggest that availability demands and accessibility stress not necessarily coincide with actual frequency of use.

Reports in the media claim nightly disturbances by mobile phone calls or messages to be a menace for today's adolescents. This may be the case among younger persons, but was not as obvious in our group of young adults, with only few being woken up regularly. However, there were cross-sectional associations between being awakened a few times or more during the past month and all mental health outcomes (no clear prospective effect).

It has been suggested that mobile phone use enhances social support [12,37], but, in our study, high frequency of use had little or no association with perceived access to social support in private life.

Quite a few participants reported subjective overuse which could indicate possible addiction to the mobile phone or its functions. Addictions can consist of excessive behaviors of all types, and some factors can be argued to be present in all types of addictions (e.g., salience, tolerance, withdrawal, conflict, and relapse) [17]. The most common symptom of problem mobile phone use among adolescents in a study by Yen et al [24] was "withdrawal symptoms without cellular phone use". Furthermore, impulsivity, especially urgency, has been related to mobile phone dependency, and feeling compelled to provide for needs as soon as possible has been suggested to increase the likelihood of using the mobile phone in a destructive way, for example when prohibited [15]. There is also the risk for addiction through gambling on mobile phones [23], which could be detrimental since the mobile phone enables gambling without time or space restrictions.

### Methodological considerations

We know little about what time span may be relevant when assessing possible effects of the exposure on mental health, and whether concurrent, short-term, or long-term exposure and effects are of interest. We have data from baseline and follow-up after 1 year, making it possible only to perform either cross-sectional analysis (so that causal inferences cannot be made) or prospective analysis with a 1-year latency period that could be considered rather long. The exposure during the latency period is not known, and the same applies to the mental health outcomes, concerning symptoms that are common in the population and that could appear and disappear in the latency period. Consequently, it is difficult to draw clear inferences about the effect of the exposure on the outcomes within the study design.

Using a questionnaire to collect information on exposure as well as health aspects poses several limitations. It is important to emphasize that the study concerns subjective symptom-reports and not actual mental disorders or diagnoses. The prevalence of reported depressive symptoms was alarmingly high in our study group. The suggested procedure that it is sufficient if one of the two PRIME-MD depressive items is confirmed in screening for depression [32,33] proposes that about 20% of the study group would be clinically depressed (positive predictive value of 33% [33]). The prevalence of depression is most likely lower in our population than in primary care populations as, for example, the 1-month prevalence of depression among Finnish young adults (20-24 years of age) was 9.6% [38]. Hence, the instrument seems too sensitive for our population, and we chose to analyze one-item and two-item responses as separate outcomes, with the expectation that the two-item outcome has higher specificity than the suggested procedure.

Recall bias and recall difficulties are most certainly present in the study, with, for example, difficulties to correctly specify the average number of calls and messages sent and received per day over the past month. Furthermore, when merging calls and SMS messages into one variable (*mobile phone use) *we lose information about specific exposure. Also, while the *high *and *low *categories are distinct from each other, the *medium *category overlaps to some extent with the *high *and *low *categories, which means that, in some instances, individuals in the *medium *category may in fact have had a higher exposure (number of calls and SMS messages) than some individuals in the *high *category, or lower than some in the *low *category. There is a risk that misclassifications obscure results.

We have limited our study to psychosocial aspects of mobile phone use. Possible biophysical pathways due to exposure to electromagnetic fields have not been considered. Furthermore, there might be factors, e.g. individual factors or personality traits, not accounted for in our study, which co-varies with exposure variables and are "true" pathways to mental health problems. This could particularly be the case concerning *accessibility stress *which had no association with *availability demands *and low association with actual frequency of use, but yet seemed to be the greatest risk factor among the mobile phone variables for developing mental health symptoms.

The study suffered from a high drop-out rate, which is fairly common when performing studies via questionnaires in the general population. The young adult population is probably especially difficult to recruit because more often than in another age group, their life situation undergoes drastic changes, including moving more often and therefore being more difficult to reach. The drop-out analysis shows that especially women and native-born Swedes are overrepresented in the data. Earlier studies, e.g. [13,14,21], have indicated gender differences in mobile phone usage, therefore gender-specific analyses were performed. However, the results of the analyses were strikingly similar for men and women in the present study. There is probably a healthy participant selection bias, and there is also an indication of bias towards lower mobile phone exposure, which could affect results in cross-sectional analyses but should have less influence in the prospective analyses. Even though the study group is more representative in comparison to studies among only college and university students, caution must be used when generalizing the results to the general population of young adults.

### Implications

The place of mobile phones as a technology distinct from landline phones on the one hand, and from computers on the other, is declining, as mobile phones increasingly are taking the place of stationary phones and at the same time are approaching computers in function. Therefore, defining the exposure becomes difficult as technology and possible uses are developing and changing rather swiftly. The use of mobile phones puts high demands on the individual's own capacity to set limits for use and accessibility. Norms on how to use mobile phones are set in interaction with others. If a young person thinks that "all others" are available at all times, he/she might feel stress if not available. Attitudes are probably an important factor to focus in prevention strategies. This could include information to children, adolescents, and young adults about the importance of sleep and recovery, and the advice to set limits for accessibility (i.e., turn off the phone) at certain times such as at nighttime, when needing to focus or rest, or when others need to focus or rest. Furthermore, shifts in attitude could also include limiting your demands and expectations on others' availability, i.e., not expecting others to be available at all times. In our study, a clear risk factor for reporting mental health symptoms was to perceive the accessibility offered by mobile phones as stressful. Thus, actually perceiving something as a "problem" could indicate a more general problem, and could serve as a warning signal for taking measures to preclude constant accessibility and overuse.

## Conclusions

There were cross-sectional and prospective associations between mobile phone variables and mental health outcomes among the young adults. High frequency of mobile phone use at baseline was a risk factor for reporting sleep disturbances and symptoms of depression for the men and symptoms of depression for the women at 1-year follow-up. The risk for reporting mental health symptoms at follow-up was greatest among those who had reported that they perceived the accessibility via mobile phones to be stressful. Public health prevention strategies focusing on attitudes could include information and advice, helping young adults to set limits for their own and others' accessibility by mobile phone.

## Competing interests

The authors declare that they have no competing interests.

## Authors' contributions

ST, AH, and MH designed the study. ST performed the data analysis and wrote the manuscript. AH and MH supervised the data analysis, and discussed and contributed to the manuscript. All authors have read and approved the final manuscript.

## Pre-publication history

The pre-publication history for this paper can be accessed here:

http://www.biomedcentral.com/1471-2458/11/66/prepub
